# Supplementary material for: ProteinShader: illustrative rendering of macromolecules
Source: BMC Struct Biol. 2009 Mar 30;9:19. doi: 10.1186/1472-6807-9-19 (PMC2672931; doi:10.1186/1472-6807-9-19)
Supplement: Additional file 1 — ProteinShader program without source code. This compressed file contains the complete ProteinShader program including associated libraries, but no source code. A README.txt file gives an overview of the ProteinShader distribution, and the index.html file in the help subdirectory has directions on getting started with the program as well as a set of tutorials. [file 1472-6807-9-19-S1.zip › ProteinShader-beta-0_9_4-binary/help/api/org/proteinshader/graphics/exceptions/class-use/TextureException.html]

Uses of Class org.proteinshader.graphics.exceptions.TextureException (ProteinShader API)


|  |  |  |
| --- | --- | --- |
| **Overview** \| **Package** \| **Class** \| **Use** \| **Tree** \| **Deprecated** \| **Index** \| **Help** | |  |
| PREV   NEXT | **FRAMES**    **NO FRAMES**     **All Classes** |  |


---


## **Uses of Class org.proteinshader.graphics.exceptions.TextureException**

| Packages that use TextureException | |
| --- | --- |
| **org.proteinshader.graphics.adapter** | Holds the StructureToGraphics class, which is used to manage the use of the drawing classes and OpenGL display lists. |
| **org.proteinshader.graphics.textures** | Holds the classes needed for creating and managing OpenGL texture objects. |

Uses of TextureException in org.proteinshader.graphics.adapter

| Methods in org.proteinshader.graphics.adapter that throw TextureException | |
| --- | --- |
| `void` | `StructureToGraphics.loadTextures(GL gl)`             Loads the default texture objects needed for text labels and real-time halftoning. |

Uses of TextureException in org.proteinshader.graphics.textures

| Methods in org.proteinshader.graphics.textures that return types with arguments of type TextureException | |
| --- | --- |
| `List<TextureException>` | `TextureFactory.getErrors()`             Returns a list with any exceptions that occurred the last time that createTextures() was called. |

| Methods in org.proteinshader.graphics.textures that throw TextureException | |
| --- | --- |
| `List<NamePair>` | `ConfigReader.readConfigFile(File file)`             Reads a configuration file to obtain a list of menu names with a matching filename for each menu name. |
| `List<NamePair>` | `EqualSignConfigReader.readConfigFile(File file)`             Reads a configuration file to obtain a list of menu names with a matching filename for each menu name. |
| `List<NamePair>` | `ConfigReader.readConfigFile(String filename)`             Reads a configuration file to obtain a list of menu names with a matching filename for each menu name. |
| `List<NamePair>` | `EqualSignConfigReader.readConfigFile(String filename)`             Reads a configuration file to obtain a list of menu names with a matching filename for each menu name. |
| `void` | `TextureManager.readTextureFiles(GL gl)`             Calls on the TextureFactory to read the texture files specified in the texture config file. |

---


|  |  |  |
| --- | --- | --- |
| **Overview** \| **Package** \| **Class** \| **Use** \| **Tree** \| **Deprecated** \| **Index** \| **Help** | |  |
| PREV   NEXT | **FRAMES**    **NO FRAMES**     **All Classes** |  |


---

# *Copyright © 2007-2008*
